# Stability Constants of Mixed Ligand Complexes of Nickel(II) with Adenine and Some Amino Acids

**DOI:** 10.1155/2015/374782

**Published:** 2015-12-30

**Authors:** Naciye Türkel

**Affiliations:** Faculty of Arts and Sciences, Department of Chemistry, Uludağ University, 16059 Bursa, Turkey

## Abstract

Nickel is one of the essential trace elements found in biological systems. It is mostly found in nickel-based enzymes as an essential cofactor. It forms coordination complexes with amino acids within enzymes. Nickel is also present in nucleic acids, though its function in DNA or RNA is still not clearly understood. In this study, complex formation tendencies of Ni(II) with adenine and certain L-amino acids such as aspartic acid, glutamic acid, asparagine, leucine, phenylalanine, and tryptophan were investigated in an aqueous medium. Potentiometric equilibrium measurements showed that both binary and ternary complexes of Ni(II) form with adenine and the above-mentioned L-amino acids. Ternary complexes of Ni(II)-adenine-L-amino acids are formed by stepwise mechanisms. Relative stabilities of the ternary complexes are compared with those of the corresponding binary complexes in terms of Δlog_10_⁡*K*, log_10_⁡*X*, and % RS values. It was shown that the most stable ternary complex is Ni(II):Ade:L-Asn while the weakest one is Ni(II):Ade:L-Phe in aqueous solution used in this research. In addition, results of this research clearly show that various binary and ternary type Ni(II) complexes are formed in different concentrations as a function of pH in aqueous solution.

## 1. Introduction

Metal ions form different complexes with various biological macromolecules or with their synthetic derivatives [1]. Nickel is one of the trace elements found in certain enzymes as cofactor [2]. Interestingly, seven out of the eight nickel-based enzymes produce or use gases in their reactions. These gases are CO, CO_2_, CH_4_, H_2_, and NH_3_ [2]. Efficacy and the specificity of nickel-based enzymes depend upon the exact coordination of nickel atom within the active site of the enzyme [3]. Both the coordination and the stability of nickel atom within the active sites of Ni-based enzymes are affected by the amino acid residues and their side chains within the active sites of those enzymes. For example, in the nickel-based superoxide dismutase (Ni-SOD) enzyme, single nickel atom is coordinated via sulfur side chains of two cysteines together with the two atoms of nitrogen from peptide backbone [4].

Metal ions also affect the structure of DNA and RNA within the cell. Metal ions, such as Mg^2+^, Ca^2+^, Mn^2+^, Cu^2+^, Zn^2+^, Cd^2+^, and Pb^2+^, can bind and distort the structure of double stranded DNA and even can form triplex or tetraplex DNA structures [5]. For example, it is a well known fact that the K^+^ ions form tetraplex DNA when they bind to Guanine rich region of chromatins [5, 6]. These types of abnormal structures on DNA may cause detrimental effects such as triplet expansion diseases [7]. It has been shown that the Schiff base complexes of Ni(II), Cu(II), and Zn(II) can bind to DNA with very high affinity in aqueous solutions [8, 9]. Schiff base complexes of these metal ions can be used as antimicrobial complexes since they can inhibit the growth of pathogenic bacteria [8, 9]. Moreover, coordination complexes of certain metal ions, including Ni(II) complexes, have a great potential to be “catalytic metallodrugs” for selective activation or degradation of target biological molecules like viral RNAs [10].

Most of the studies on the stability and the coordination of nickel in enzymes or in nucleic acids has been done using whole enzymes and large nucleic acid molecules [4, 5, 11]. However, stability and the coordination of nickel within the metal centers of the enzymes are influenced by the tri-dimensional structure and also by the substrate binding sites of these type enzymes. In addition, nickel can be found with other metal ions, such as iron, in certain Ni-based enzymes [2]. Furthermore, super coiled structures of nucleic acids may also interfere with stable interactions of Ni(II) with individual bases of DNA or RNA [5].

In a past study, Krause et al. examined the interaction and the stability of Ni(II) with amino acids with dipeptide which is composed of asparagine-cysteine-cysteine (also known as NCC peptide) instead of whole enzyme nickel superoxide dismutase (Ni-SOD) [12]. In that study, it has been shown that coordination of Ni(II) within the active site of Ni-SOD can be mimicked by NCC tripeptide. Biological systems have a large set of target molecules for metal ion interactions. In addition to nickel, metal ions such as sodium, potassium, magnesium, calcium, manganese, iron, cobalt, copper, zinc, and molybdenum are also present within different types of living cells and they compete with each other to form various metal-ligand complexes with biological macromolecules [1, 13]. In certain cases, metal ions should form relatively weak interactions with proteins as in the cases of metal ion transporters [14]. On the contrary, metal ions interact rather strongly with the amino acids in the active sites of metalloenzymes [14]. Hence, it is important to analyze the strength of the metal-ligand interactions in aqueous solution with mixed ligand systems.

In this study, stabilities of the binary and ternary complexes of nickel with adenine and free amino acids L-Asp, L-Glu, L-Asn, L-Leu, L-Phe, and L-Trp were investigated in aqueous solution by potentiometric methods. Results of this study indicate that stabilities of binary and ternary complexes of Ni(II) with these ligands depend on the pH of the aqueous solution and the structure of the L-amino acids. Results of this research can be used to evaluate the interactions of nickel ions with DNA bases and L-amino acids in aqueous solution in the presence of mixed ligands at molecular level.

## 2. Material and Methods

### 2.1. Materials

Stock solution of the nickel (~0.01 mol·L^−1^) used in the study was prepared from the nitrate salt of (Ni(NO_3_)_2_·6H_2_O). This stock solution was acidified with HCl to prevent the hydrolysis of the metal, and concentration of the acid solution was determined through Gran method potentiometrically [15]. Concentration of the Ni(II) solution was determined by complexometric EDTA titration [15–17]. Potassium hydrogen phthalate was used to standardize carbonate-free sodium hydroxide (titrant, prepared in 0.1 mol·L^−1^ KNO_3_ solution) potentiometrically [18, 19]. The amino acids (L-Asp (98%), L-Glu (99.5%), L-Asn (98%), L-Leu (98%), L-Phe (98%), and L-Trp (98%)) and adenine (99%) were used as received. An HCl solution (0.1 mol·L^−1^) was prepared and used after standardization as described [20]. All of the solutions used during the experiments were prepared freshly in ultra pure water having a resistivity of 18.3 mol·L^−1^Ω cm. Samples of all of the aqueous solutions were prepared gravimetrically.

### 2.2. pH-Metric Measurements

A special glass vessel for potentiometric titrations device was used as explained in previous studies [21]. This titration system has a double wall, with entries for combined glass electrode (Schott), nitrogen, and base from the burette (TL 7000-M2/20 automatic titrator). The emf measurements were carried out with the cited automatic titrator every 4 minutes. Temperature inside the cell was kept constant at (25.0 ± 0.1)°C, through circulation of water from an external thermostat (RW-0525G). Each of the investigated solutions was thermostated at the necessary temperature with an accuracy of ±0.1°C, and the solutions were left to stand at this temperature for 15 min prior to titration. Calibration of the combined glass electrode was performed in both acidic and alkaline regions by titrating 0.01 mol L^−1^ hydrochloric acid with standard sodium hydroxide prior to each titration to read the hydrogen ion concentration directly so that the p[H] is defined as −log_10_⁡[H] [22]. The emf values (*E*) depend on [H^+^] according to *E* = *E* ^*o*^ + *s*log_10_[H^+^] + *J*_H_[H^+^] + *J*_OH_[OH^−^], where *J*_H_ and *J*_OH_ are fitting parameters in acidic and alkaline media in order to correct experimental errors. These errors arise mainly from the liquid junction and the alkaline and acidic errors of the glass electrode. The autoprotolysis constant of water, p*K*_w_ for the aqueous system, defined as −log_10_⁡([H^+^]+[OH^−^]) at the ionic strength used, was found to be ~13.85.

Total volume in all potentiometric titrations was adjusted to 50 mL. The following reaction mixtures (described from reactions (a) to (i)) containing proton and/or Ni(II) nitrate and the ligands at different ratios (1 : 1), (1 : 2), and (1 : 3) in binary systems, and (1 : 1 : 1) in ternary systems, were titrated through incremental additions of carbonate-free and standard NaOH:

- HCl (0.1 mol·L^−1^, 5.0 mL) + (1.0 mol·L^−1^, 5.0 mL KNO_3_) + H_2_O (for cell calibration).
- HCl (0.1 mol·L^−1^, 2.0 mL) + (1.0 mol·L^−1^, 5.0 mL KNO_3_) + H_2_O.
- Solution (b) + Ade (A, 0.01 mmol) (for determining the protonation constants of Ade).
- Solution (b) + amino acids (B, 0.01 mmol) (for determining the protonation constants of amino acids).
- Solution (b) + Ade (A, 0.01 mmol) + Ni(II) (0.01 mol·L^−1^, 10.0 mL) (for determining the stability constants of the NiA binary complexes).
- Solution (b) + Ade (A, 0.02 mmol) + Ni(II) (0.01 mol·L^−1^ 10.0 mL) (for determining the stability constants of the NiA_2_ binary complexes).
- Solution (b) + amino acids (B, 0.01 mmol) + Ni(II) (0.01 mol·L^−1^, 10.0 mL) (for determining the stability constants of the NiB binary complexes).
- Solution (b) + amino acids (B, 0.02 mmol) + Ni(II) (0.01 mol·L^−1^, 10.0 mL) (for determining the stability constants of the NiB_2_ binary complexes).
- Solution (b) + Ade (A, 0.01 mmol) + amino acids (B, 0.01 mmol) + Ni(II) (0.01 mol·L^−1^, 10.0 mL) (for determining the stability constants of the NiAB (1 : 1 : 1) ternary complexes).

Titrations for each set were conducted at least four times in order to check the reproducibility of the data. These systems were evaluated in the pH range of 1.5–10.5, since the changes in ionic strength and the p[H]-dependence of the junction potentials could affect the accuracy.

## 3. Results and Discussion

### 3.1. Proton-Ligand Equilibria

Nickel can interact with various amino acids and their side chains within the active sites of nickel-based enzymes [2]. To analyze the stabilities of nickel complexes with L-amino acids in aqueous solution, six different types of L-amino acids were selected based on the biochemical features of their side chains. These L-amino acids are L-Asp, L-Glu, which have negatively charged R-groups, and L-Asn that has an uncharged polar R-group. L-Leu, L-Phe, and L-Trp were selected as an example to apolar R-groups containing L-amino acids. Adenine was selected for sample base due to its significant role in the ATP and ADP in the biological reactions.

**Figure 1:**
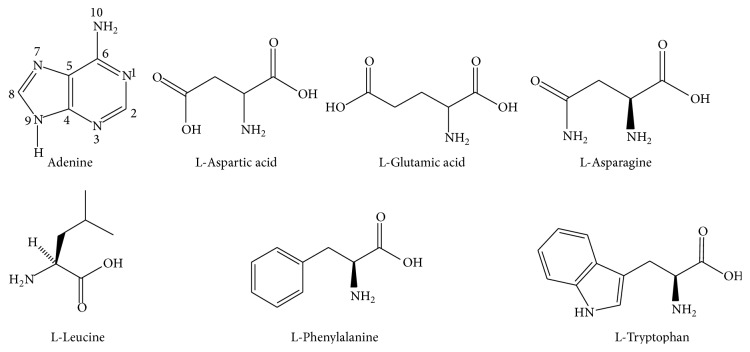
Structures of the ligands in this study.

Protonation constants of adenine and amino acids (L-Asp, L-Glu, L-Asn, L-Leu, L-Phe, and L-Trp) (open formulae of the ligands selected for this study are provided in Figure 1) examined in this study were determined by potentiometric method using the computer program named “BEST” developed through Martell and Motekaitis at 0.1 mol·L^−1^ KNO_3_ ionic medium and 25.0°C [23]. Protonation constants of all of the ligands determined in this study were listed in Table 1. It is clear that all protonation constants (log_10_⁡*K*_1_, log_10_⁡*K*_2_, and log_10_⁡*K*_3_) of Ade and L-amino acids identified in this research are very close to previously reported values for those ligands (Table 1) [24–32].

### 3.2. Nickel(II)-Adenine Equilibria

**Figure 2:**
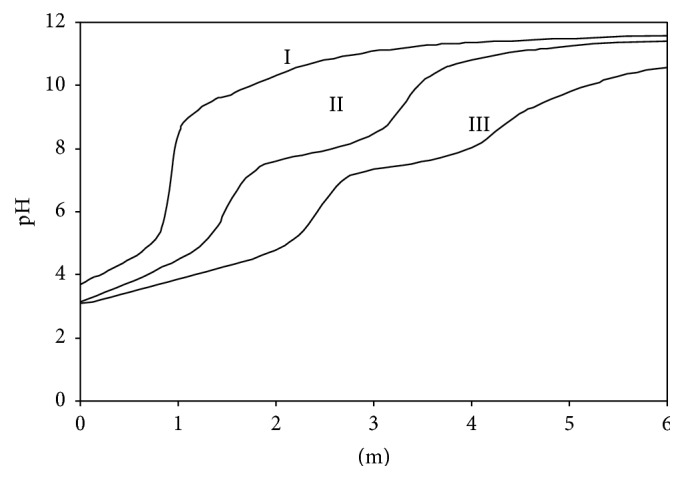
Potentiometric titration curves of binary complexes (*I* = 0.1 mol·L^−1^ KNO_3_ at 25.0 ± 0.1°C). Curve I: adenine alone, curve II: Ni(II):adenine (1 : 1), and curve III: Ni(II):adenine (1 : 2).

Adenine was titrated alone and in the presence of Ni(II) ion potentiometrically. Potentiometric titration curve of the Ni(II):Ade system was given in Figure 2, curve II. The decrease in the pH value of the reaction mixture indicated that Ni(II):Ade complexes form at 1 : 1 and 1 : 2 mol ratios as seen in Figure 2, curve II for (1 : 1) and curve III for (1 : 2) binary complexes of Ni(II):Ade, respectively. Hamada et al. demonstrated in their work that large metal ions (such as Cd(II) or Hg(II) with ionic radius = 95 and 102 pm, resp.) will coordinate in a chelate fashion between the N(3) and N(9) in adenine. Smaller metal ions such as Zn(II) (74 pm) will fit in the smaller chelate ring between the N(7) and N(10) in adenine as shown in Figure 1 [33]. The stability constants of these complexes, which were formed, were calculated (according to (1) and (2)) at 0.1 mol·L^−1^ KNO_3_ ionic medium and 25.0°C temperature with BEST computer program and given in Table 2. From these results, it was seen that the results presented in the previous literature were coherent with the values of this research [34, 35].

In the following reactions and equations, Ni(II) metal ion and adenine (A:Ade) are present in (1 : 1) and (1 : 2) stoichiometry, respectively. The equilibrium reactions and equations (1) and (2) are given for binary complex systems which form in these situations.

For A (Ade),

(1) Ni+A⇌NiAβ1=βNiNiA=NiANiA

(2) Ni+2A⇌NiA2β2=βNiNiA2=NiA22NiA2

(charges are omitted for the intention of clarity).

### 3.3. Nickel(II) Amino Acids Equilibria

**Figure 3:**
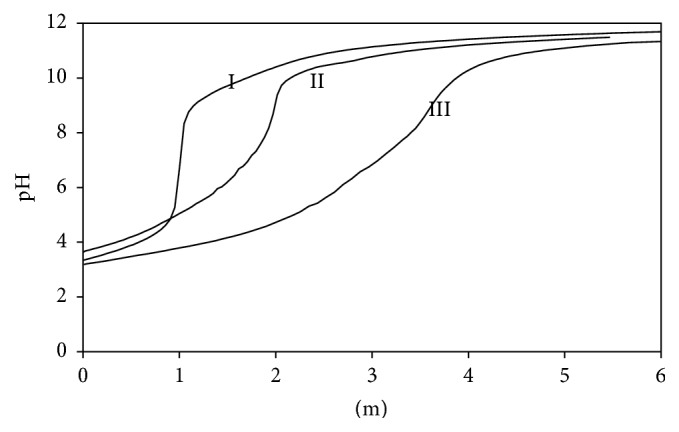
Potentiometric titration curves of binary complexes (*I* = 0.1 mol·L^−1^ KNO_3_ at 25.0 ± 0.1°C). Curve I: L-aspartic acid alone, curve II: Ni(II):L-aspartic acid (1 : 1), and curve III: Ni(II):L-aspartic acid (1 : 2).

**Figure 4:**
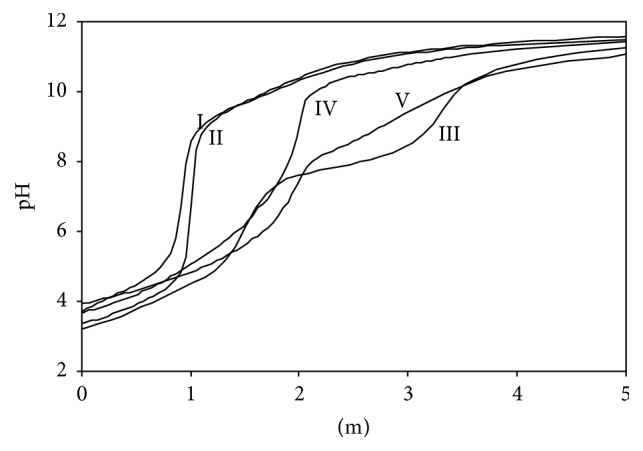
Potentiometric titration curves of binary (1 : 1) and ternary (1 : 1 : 1) complexes (*I* = 0.1 mol·L^−1^ KNO_3_ at 25.0 ± 0.1°C). Curve I: adenine alone, curve II: L-aspartic acid alone, curve III: Ni(II):adenine (1 : 1), curve IV: Ni(II):L-aspartic acid, and curve V: Ni(II):adenine:L-aspartic acid (1 : 1 : 1).

Potentiometric titrations of each amino acid selected for this study were performed alone, and also in the presence of Ni(II) ion in aqueous solution in (1 : 1) to (1 : 3) stoichiometry, at 0.1 mol·L^−1^ KNO_3_ ionic medium, 25.0°C, in nitrogen atmosphere. Instead of giving potentiometric titration curves for all Ni(II):L-amino acid reactions, only Ni(II):L-aspartic acid curves were given for simplicity (Figure 3). In Figures 2–4, m in *x*-axis represents mmole base/mmole metal or mmole base/mmole ligand. In order to calculate the stability constants of Ni(II) ion with L-amino acids used in this study, protonation constants given in Table 1 were used [23]. The stability constants of binary coordination complexes of Ni(II) with L-amino acids determined in this study were given in Table 2. Literature values on the stability constants of binary complexes of Ni(II):L-amino acids were also listed in Table 2 for comparison [36–41]. It can be seen that Ni(II) ion forms coordination complexes at 1 : 1 and 1 : 2 mole ratios with L-Asp and L-Leu, respectively. It is also clear that Ni(II) ion forms 1 : 1, 1 : 2, and 1 : 3 coordination compounds with L-Glu, L-Asn, L-Phe, and L-Trp (Table 2) [36–41].

The following equilibrium reactions and equations ((3)–(5)) are given for binary complex systems which are thought that Ni(II) metal ion and each of the amino acids (B) form in (1 : 1), (1 : 2), and/or (1 : 3) stoichiometry:

(3) Ni+B⇌NiBβ1=βNiNiB=NiBNiB

(4) Ni+2B⇌NiB2β2=βNiNiB2=NiB2NiB2

(5) Ni+3B⇌NiB3β3=βNiNiB3=NiB3NiB3

(charges are omitted for the intention of clarity).

### 3.4. Ternary Complexes of Ni(II) Ion

The stability constants of the ternary complexes of Ni(II) ions that form between Ni(II):Ade:L-amino acids at 1 : 1 : 1 mole ratio were calculated from the potentiometric titration results. As in the binary complexes, instead of giving the graphical results for all of the ternary complexes, potentiometric titration curves for the ternary complex of Ni(II):Ade:L-asp were given as an example for simplicity (Figure 4). The stability constants calculated according to (6) by the BEST computer program are also provided in Table 3:

(6) $$\begin{array}{c} Ni+A+B⇌NiAB\;\beta_{NiAB}={\beta^{Ni}}_{NiAB}=\frac{\left[NiAB\right]}{\left[Ni\right]\left[A\right]\left[B\right]} \end{array}$$

(A: adenine, B: amino acid).

### 3.5. Distribution Diagrams

**Figure 5:**
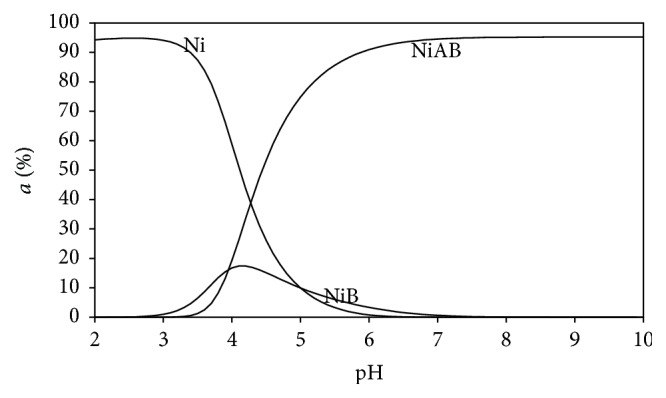
Distribution diagram of the species in the (1 : 1 : 1) Ni(II):adenine(A):L-aspartic acid(B) ternary system.

**Figure 6:**
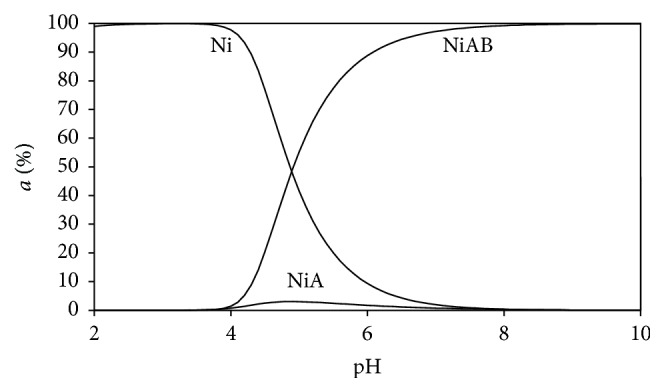
Distribution diagram of the species in the (1 : 1 : 1) Ni(II):adenine(A):L-glutamic acid(B) ternary system.

**Figure 7:**
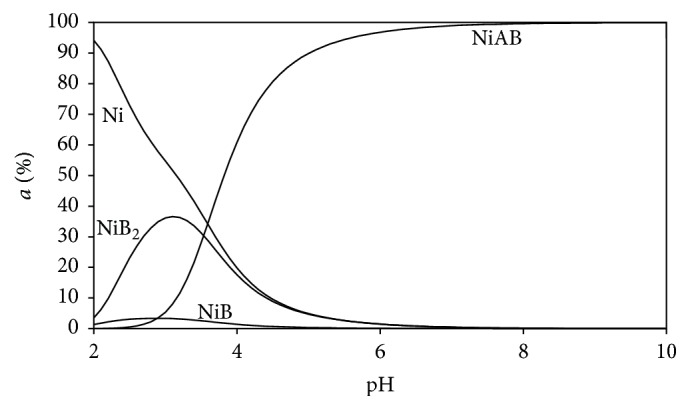
Distribution diagram of the species in the (1 : 1 : 1) Ni(II):adenine(A):L-asparagine(B) ternary system.

**Figure 8:**
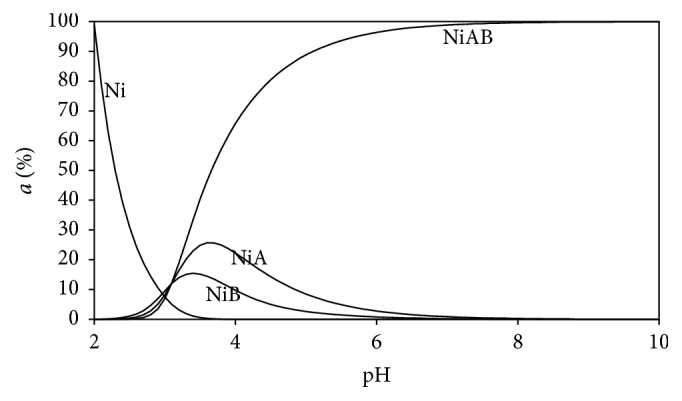
Distribution diagram of the species in the (1 : 1 : 1) Ni(II):adenine(A):L-leucine(B) ternary system.

**Figure 9:**
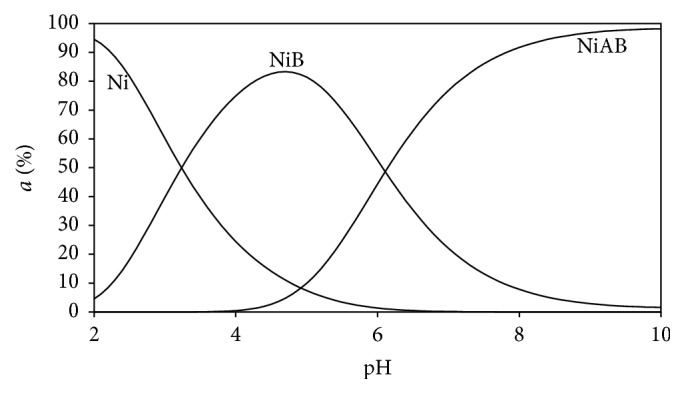
Distribution diagram of the species in the (1 : 1 : 1) Ni(II):adenine(A):L-Phenylalanine(B) ternary system.

**Figure 10:**
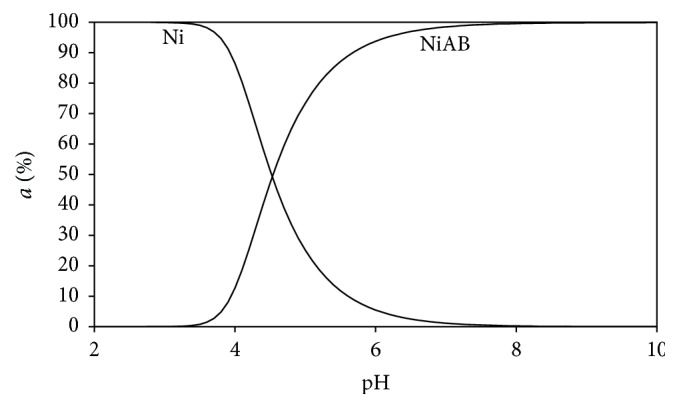
Distribution diagram of the species in the (1 : 1 : 1) Ni(II):adenine(A):tryptophan(B) ternary system.

Distribution diagrams of the all Ni(II) ternary systems were drawn by the SPE computer program [23]. Stability constants of binary and ternary complexes given in Tables 2 and 3 were used to see the changes of distributions of all types in mixed (ternary) systems as a function of the pH (Figures 5–10). As we see in potentiometric titration curves, the main feature of each of the distribution diagrams shown in Figures 5–10 is that NiAB ternary complexes form in pH = 2.0–10.0 range.

Ni(II):L-Asp binary complex begins to form at pH = 3. It reaches maximum level (15%) at pH = 4.1, and Ni(II):L-Asp complex disappears completely at pH = 7 in this aqueous solution (shown as NiB in Figure 5). Ni(II):Ade:L-Asp ternary complex begins to form at pH = 3. It reaches its maximum level (95%) at pH = 6 (shown as NiAB in Figure 5). Ni(II):Ade:L-Asp ternary complex remains stable as dominant complex in this aqueous solution between pH 6 and pH 10.

Ni(II):Ade binary complex forms at very low levels at pH = 4–8 (Figure 6). At the same pH levels, Ni(II):Ade:L-Glu ternary complexes begin to form. The level of this complex reaches its maximum level (100%) at pH = 7 (shown as NiAB in Figure 6).

Ni(II):Ade:L-Asn ternary complex formation reactions were also investigated in aqueous solution. As it can be seen in Figure 7, Ni(II):L-Asn binary complexes 1 : 1 and 1 : 2 form at pH 2–6. In addition, it may be seen that Ni(II):Ade:L-Asn complex begins to form at pH = 2, and it reaches its maximum level (99%) at pH 6.

The diagrams drawn for the Ni(II):Ade:L-Leu ternary system showed a different behavior from other ternary systems analyzed in this research (Figure 8). It is clearly seen that binary (Ni(II):Ade and Ni(II):Leu) and ternary (Ni(II):Ade:L-Leu) complex begin to form at the same time and at the same pH level (pH = 2.5). Moreover, Ni(II):Ade:L-Leu ternary complex is present as single dominant coordination species at pH = 6–10.

Ni(II):Ade:L-Phe ternary complex formation also shows different trends from the other complexes of Ni(II) (Figure 9). Ni(II):L-Phe binary complex begins to form at pH = 2, and it reaches its maximum level (82%) at pH = 5. Ni(II):L-Phe binary complex begins to dissociate gradually at pH = 5, and it completely disappears at pH = 10. In this Ni(II), Ade, L-Phe mixture, Ni(II):Ade:L-Phe type ternary complex formation begins at pH = 4 and it reaches its maximum level at pH = 8.5.

Unexpectedly, in Ni(II), Ade, L-Trp system, binary complex of Ni(II) is not detectable, since the Ni(II):Ade:L-Trp ternary complex dominates the reaction in this aqueous solution (Figure 10). Formation of Ni(II):Ade:L-Trp ternary complex begins at pH = 3.8, and it reaches its maximum level at pH = 7.

log_10_⁡*K* ^NiA^_NiAB_ and log_10_⁡*K* ^NiB^_NiAB_ stability constants were compared with each other in order to decide which one of the ligands was contributing to formation of the mixed ligand complexes, and which one is acting as the primary or secondary ligand. For this purpose, the following equations were used:

(7) log10⁡βNiABlog10⁡βNiNiAB=log10⁡KNiNiA+log10⁡KNiANiAB,log10⁡KNiANiABlog10⁡βNiNiAB−log10⁡KNiNiA,log10⁡βNiABlog10⁡βNiNiAB=log10⁡KNiNiB+log10⁡KCBuNiAB,log10⁡KNiBNiABlog10⁡βNiNiAB−log10⁡KNiNiB.

log_10_⁡*K* ^NiA^_NiAB_ and log_10_⁡*K* ^NiB^_NiAB_ constants were calculated for each mixed ligand system as shown in Table 3. It can be seen that adenine acts as the primary ligand particularly in the Ni(II):Ade:L-Glu, Ni(II):Ade:L-Leu, and Ni(II):Ade:L-Trp systems and amino acids act as the primary ligand in Ni(II):Ade:L-Asp, Ni(II):Ade:L-Asn, and Ni(II):Ade:L-Phe systems.

It is quite difficult to decide the stability of the mixed ligand complexes just by taking the stability constant as the basis. Therefore, stability of mixed ligand complexes should be evaluated together with the stability of binary complexes [25]. For this purpose, the differences (Δlog_10_⁡*K*) between the stabilities of the Ni(II):A:B and Ni(II):A were compared. Δlog_10_⁡*K* is the value that characterizes the coordination tendency of a second ligand to Ni(II):A complexes associated with Ni(II). The equilibrium reaction and equation represented by (8) were calculated by the equation shown in (9). Thus,

(8) NiA+NiBNiAB+Ni

(9) Δlog10⁡Klog10⁡KNiANiAB−log10⁡KNiNiB=log10⁡KNiBNiAB−log10⁡KNiNiA.

Δlog_10_⁡*K* values for Ni(II):A:B mixed ligand complexes were calculated by using (8) and (9) and the values found were given in Table 3. When Δlog_10_⁡*K* values were examined, it was observed that Δlog_10_⁡*K* values obtained in all systems except the Ni(II):Ade:L-Phe system were positive. This shows that equilibrium shown in (8) (except Ni(II):Ade:L-Phe) tends to form the ternary complex of NiAB. The results show that ternary complexes are more stable than the binary complexes analyzed in this research. However, it was also seen that Ni(II):Ade:L-Phe ternary complex is less stable than the binary system (Ni(II):Ade or Ni(II):L-Phe).

The second way of characterizing the formation tendency of the Ni(II):mixed ligand complexes is log_10_⁡*X* (nonproportional dissociation constant) values [42]. This constant equilibrium expression is calculated by equilibrium equations:

(10) NiA2+NiB2⇌2NiABX=NiAB2NiA2·NiB2

(11) log10⁡X=2×log10⁡βNiNiAB−log10⁡βNiNiA2+log10⁡βNiNiB2.

When the log_10_⁡*X*'s values given in Table 3 are examined, it can be seen that, in each of the mixed ligand complexes, the log_10_⁡*X* value is bigger than the statistical value of 0.6 (*X* = 4). This shows that the formation of mixed ligand complexes is more prevalent than binary complexes. When the Ni(II):Ade:L-Asp (log_10_⁡*X* = 0.96), Ni(II):Ade:L-Glu (log_10_⁡*X* = 0.93), Ni(II):Ade:L-Asn (log_10_⁡*X* = 0.85), Ni(II):Ade:L-Leu (log_10_⁡*X* = 0.90), Ni(II):Ade:L-Phe (log_10_⁡*X* = 0.93), and Ni(II):Ade:L-Tryp (log_10_⁡*X* = 1.01) values are examined, it can be seen that ternary complexes, especially for Ni(II):Ade:L-Trp system, form at high percentages in all the distribution diagrams (Figure 10).

Another parameter, which is percent relative stabilization (% RS) for quantifying the stability of a ternary complex, may be defined as [43]:

(12) $$\begin{array}{c} \%\text{ R.S}=\left[\;\frac{\left(\log_{10}{K^{NiA}}_{NiAB}-\log_{10}{K^{Ni}}_{NiB}\right)}{\log_{10}{K^{Ni}}_{NiB}}\;\right]\times 100. \end{array}$$

The values obtained agree with the Δlog_10_⁡*K* values as shown in Table 3.

When all the distribution diagrams are examined, it can be observed that both binary Ni:L-Phe and ternary Ni:Ade:L-Phe complexes were dominant only in the Ni:Ade:L-Phe system (Figure 9). This can also be confirmed by the negative values of Δlog_10_⁡*K* and % RS of this system.

## 4. Conclusions

The results of this research indicate that Ni(II) ions can form binary and ternary complexes with adenine and L-amino acids at various combinations when these compounds are present as mixed ligand systems in an aqueous solution:

(a) Our studies suggest that adenine might be coordinated to Ni(II) ion (72 pm) through the (N7) and the (N10) while above-mentioned amino acids might be coordinated through the amino nitrogen and carbonyl oxygen.
(b) The order of stability of the Ni(II) ion obtained in the mixed ligand complex systems examined in aqueous solution in this study is as follows: Ni(II):Ade:L-Phe < Ni(II):Ade:L-Glu < Ni(II):Ade:L-Leu < Ni(II):Ade:L-Trp < Ni(II):Ade:L-Asp < Ni(II):Ade:L-Asn. Regarding the Δlog_10_⁡*K* value computed for the mixed ligand complex systems, Ni(II):Ade:L-Asn (Δlog_10_⁡*K* = 4.12) had the highest values of stability. This, in turn, is in line with the stated order of stability.
(c) The fact that the negative log_10_⁡*K* and % RS values were obtained from the mixed ligand complex systems Ni(II):Ade:L-Phe (log_10_⁡*K* = −0.14, % R.S = −1.57) shows that the stability of binary complex systems is more dominant than that of the mixed ligand complex systems. When we examine Figure 9, we can see that NiB(Ni:L-Phe) binary coordination compound is dominant in pH = 2.0–10 range. However, Ni(II):Ade:L-Phe ternary coordination compound is formed after pH = 4.0, and it is the only dominant type after pH = 8.0.
(d) When we examine Table 3, we see that the highest log_10_⁡*X* = 1.01 and % RS = 72.48 values belong to the Ni(II):Ade:L-Trp ternary system. These values confirm the presence of Ni(II):Ade:L-Trp as the only dominant type in Figure 10 in the pH = 3.8–10.0 range.

## Figures and Tables

**Table 1:** The protonation constants of the ligands at 25.0 ± 0.1°C, *I* = 0.1 mol·L^−1^ KNO_3_.

	log_10_⁡*K*_1_	log_10_⁡*K*_2_	log_10_⁡*K*_3_
Ade(A)	9.60 ± 0.06	4.18 ± 0.07	
	9.60 [24]	3.90 [24]	
	9.62 [25]	4.20 [25]	
	9.92 [26]	4.19 [26]	

L-Asp(B)	9.61 ± 0.03	3.74 ± 0.03	2.00 ± 0.03
	9.64 [25]	3.70 [25]	1.98 [25]
	9.82 [27]	3.86 [27]	2.10 [27]
	9.48 [28]	3.87 [28]	2.48 [28]

L-Glu(B)	9.57 ± 0.05	4.16 ± 0.05	2.19 ± 0.05
	9.58 [25]	4.14 [25]	2.16 [25]
	9.41 [27]	4.15 [27]	2.65 [27]
	9.98 [29]	4.37 [29]	2.05 [29]

L-Asn(B)	8.78 ± 0.02	2.17 ± 0.02	
	8.73 [25]	2.16 [25]	
	8.45 [24]	1.70 [24]	

L-Leu(B)	9.52 ± 0.05	2.31 ± 0.05	
	9.56 [25]	2.34 [25]	
	9.58 [30]	2.32 [30]	

L-Phe(B)	9.14 ± 0.07	2.19 ± 0.07	
	9.10 [25]	2.18 [25]	
	9.75 [31]	2.14 [31]	

L-Trp(B)	9.38 ± 0.03	2.31 ± 0.03	
	9.33 [25]	2.28 [25]	
	9.14 [32]	2.22 [32]	

Uncertainties are expressed as ± one standard deviation.

**Table 2:** The stability constants of binary complexes at 25.0 ± 0.1°C, *I* = 0.1 mol·L^−1^ KNO_3_.

	log_10_⁡*β*_1_	log_10_⁡*β*_2_	log_10_⁡*β*_3_
Ni(II):adenine(A)	6.66 ± 0.06	12.22 ± 0.06	
	7.88 [34]		
	6.66 [35]	11.11 [35]	
Ni(II):L-Asp(B)	8.14 ± 0.06	12.70 ± 0.06	
	7.14 [36]	12.43 [36]	
	7.25 [37]		
Ni(II):L-Glu(B)	5.34 ± 0.03	10.36 ± 0.03	13.43 ± 0.03
	6.06 [38]	10.33 [38]	12.51 [38]
Ni(II):L-Asn(B)	8.02 ± 0.05	18.29 ± 0.05	25.55 ± 0.04
	8.64 [39]	17.26 [39]	25.64 [39]
Ni(II):L-Leu(B)	5.53 ± 0.04	11.78 ± 0.04	
	5.5575 [40]	9.1013 [40]	
Ni(II):L-Phe(B)	6.52 ± 0.02	10.10 ± 0.02	16.58 ± 0.02
	5.4034 [40]	9.4963 [40]	
Ni(II):L-Tryp(B)	5.45 ± 0.02	9.64 ± 0.02	13.62 ± 0.02
	5.76 [41]	10.98 [41]	15.46 [41]

Uncertainties are expressed as ± one standard deviation.

**Table 3:** The stability constants and parameters of ternary (mixed NiAB) complexes at 25.0 ± 0.1°C, *I* = 0.1 mol·L^−1^ KNO_3_. % RS is the percentage relative stabilization value, as explained in (12).

	log_10_⁡β_NiAB_	log_10_⁡*K* ^NiA^_NiAB_	log_10_⁡*K* ^NiB^_NiAB_	Δlog_10_⁡*K*	log_10_⁡*X*	% R.S
Ni(II):Ade(A):L-Asp(B)	17.01 ± 0.06	10.35	8.87	2.21	0.96	27.14
Ni(II):Ade(A):L-Glu(B)	15.57 ± 0.06	8.91	10.23	3.574	0.93	66.85
Ni(II):Ade(A):L-Asn(B)	18.80 ± 0.03	12.14	10.78	4.12	0.85	51.37
Ni(II):Ade(A):L-Leu(B)	16.01 ± 0.05	9.35	10.48	3.82	0.90	69.07
Ni(II):Ade(A):L-Phe(B)	15.43 ± 0.04	8.77	6.52	−0.14	0.93	−1.57
Ni(II):Ade(A):L-Tryp(B)	16.06 ± 0.02	9.40	10.61	3.95	1.01	72.48

Uncertainties are expressed as ± one standard deviation.
